# A Highly Sensitive Molecularly Imprinted Polymer (MIP)-Coated Microwave Glucose Sensor

**DOI:** 10.3390/s22228648

**Published:** 2022-11-09

**Authors:** Amir Hossein Omidvar, Atena Amanati Shahri, Ariana Lacorte Caniato Serrano, Jonas Gruber, Gustavo Pamplona Rehder

**Affiliations:** 1 Department of Electronic Systems, Polytechnic School, University of São Paulo, São Paulo 05508-010, Brazil; 2 Department of Fundamental Chemistry, Institute of Chemistry, University of São Paulo, São Paulo 05508-000, Brazil

**Keywords:** stub resonator, microwave sensors, molecular imprinted polymer, biosensor, glucose sensor

## Abstract

A novel, low-cost, sensitive microwave microfluidic glucose detecting biosensor incorporating molecularly imprinted polymer (MIP) is presented. The sensing device is based on a stub resonator to characterize water glucose solutions. The tip of one of the stubs is coated with MIP to increase the selectivity of the sensor and hence the sensitivity compared to the uncoated or to the coated with non-imprinted polymer (NIP) sensor. The sensor was fabricated on a FR4 substrate for low-cost purposes. In the presence of the MIP, the sensor loaded with a glucose solution ranging from 50 mg/dL to 400 mg/dL is observed to experience an absorption frequency shift of 73 MHz when the solutions flow in a microfluidic channel passing sensing area, while the lower limit of detection (LLD) of the sensor is discovered to be 2.4 ng/dL. The experimental results show a high sensitivity of 1.3 MHz/(mg/dL) in terms of absorption frequency.

## 1. Introduction

Diabetes is one of the four main noncommunicable diseases and has become one of the fastest growing diseases in the world. It will be the seventh leading cause of death worldwide by 2030 [1]. The International Diabetes Federation (IDF) has released a new report that demonstrates the global population with diabetes has reached 537 million people, increasing 16% since their previous estimation in 2019 [2].

Monitoring blood glucose level is important in managing diabetes and maintaining it in a safe range (between 80–120 mg/dL) is essential. *Biosensing* technologies are *constantly* being examined, and two types of optical [3] and electrochemical [4] biosensors are available in the market. However, electrochemical biosensors are widely used in clinical practice for the sensitive detection of glucose. The majority of these conventional glucose sensors are based on enzymatic methods adapting different approaches such as electrochemical, colorimetric, and chemiluminescence [5,6,7]. They initially utilize glucose oxidase (GOD) enzyme, based on a co-product of enzymatic oxidation of glucose molecules by tracking H_2_O_2_ to detect and measure glucose. Nevertheless, GOD can be damaged by some chemical substances and detergents or changes in its optimal environment. Indeed, those GOD-based glucose sensors are always vulnerable to deactivation due to chemical deformation and possible thermal changes during fabrication, storage, or even while using [8].

Therefore, many researchers have tried to demonstrate the feasibility of alternative sensing methods for precise and reliable glucose monitoring. Considerable studies proposed microwave sensors for glucose level detection. The capability of microwave-based sensors to non-destructively distinguish and measure the parameters inside the liquid sample volumes make it a great alternative to the invasive daily glucose monitoring techniques. Microwave biosensors also offer high sensitivity and real-time monitoring [9]. Another unique approach of these sensors is that they can perform passively (battery-less). The sensing principle is based on harvesting energy released in the environment and reflected radiation from the target under test [10].

Researchers proposed several configurations based on planar technology for resonant frequency-based glucose sensors [9]. The principle of these sensors is the correlation between the resonant frequency and the changes in the relative permittivity. The microstrip line (MLIN)-based resonators such as Split Ring Resonators (SRR) and Stub Resonators are the most widely used structures for this purpose and attract interest due to their low cost, small size, and ease of fabrication. A microstrip line-based sensor was proposed to quantify the variation of the glucose concentration by integrating the MUT in a cavity under the microstrip transmission line [11]. Stub resonators have also been studied in the past decade, and a miniature double-stub resonant-based sensor was introduced to detect the glucose concentrations of two aqueous solutions positioned over the sensing area of each stub [12]. The simple design and fabrication are the primary advantage of this technique. Yet the sensitivity of these biosensors is the main limitation and restricts their application in practical glucose monitoring. Some researchers use labels such as enzymes to enhance sensitivity. In this context, enzyme-coated SRR with different geometrical shapes were utilized. For instance, a single SRR configuration has been proposed with an enzyme-coated extended capacitive gap [13,14]. However, the insufficient stability created due to the nature of the enzymes is the main disadvantage of utilizing enzymes in monitoring glucose applications.

Therefore, technical improvements are required to increase sensitivity and selectivity while reducing size and cost. Accordingly, synthetic receptors and molecular imprinting to develop such synthetic receptors can potentially reduce and/or removing limitations of enzyme [15]. Molecular Imprinting Technology (MIT) forms artificial receptors and provides prearranged selectivity in relation to a specifically targeted molecule that is practical in various fields [16].

**Figure 1:**
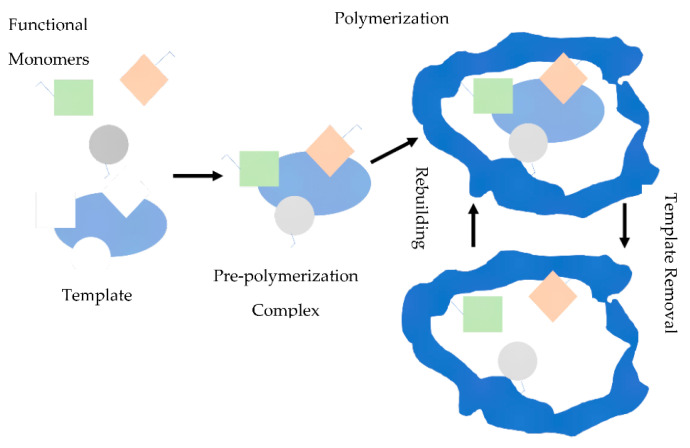
Molecular imprinting schematic illustration.

The molecularly imprinting process usually involves starting the polymerization of monomers in the presence of a template molecule that is extracted later, which leaves complementary cavities. These polymers have an affinity for the original molecule. The molecular imprinting schematic illustration is shown in Figure 1. This method has been applied practically to combine materials with molecular selectivity in past decades [17]. It is reported that molecularly imprinted polymers (MIPs) are selective for their template. It implies that when a novel MIP is made for a template, it interacts with the template selectively compared to similar compounds [18]. Thus, molecularly imprinted polymers attract considerable attention in sensor fabrication due to their excellent selectivity. Indeed, new sensors using MIPs were developed in a wide variety of applications due to their high thermal stability, reusability, and selectivity [19,20,21,22].

This paper presents a novel, simple, inexpensive, non-enzymatic, and sensitive characterization method by introducing a microfluidic integrated MIP-coated microwave biosensor capable of quantifying glucose concentration. A glucose imprinted polymer film is synthesized and applied as a selective coating on a stub resonator, and a microfluidic system is integrated on the top of the sensing area to control the flow of the liquid for an accurate dielectric measurement. Changes in resonant frequency due to glucose variation in the transmitted signal are analyzed.

## 2. Materials and Methods

### 2.1. Sensor Design

**Figure 2:**
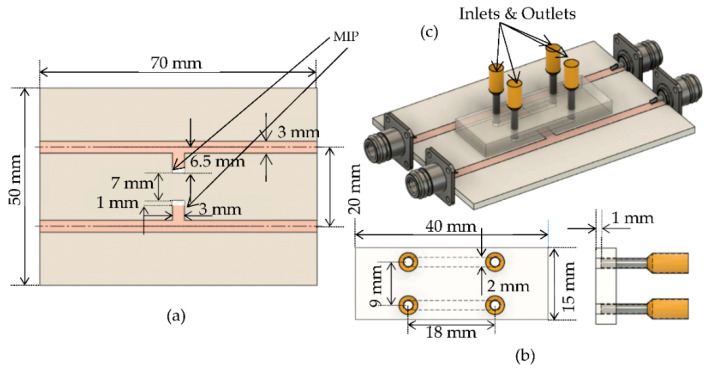
The designed MIP-coated microwave-based stub biosensor (**a**) Top view of the biosensor (**b**) Top view and side view of the microfluidic system with the inlets and outlets (**c**) View of the assembled biosensor.

The model of the proposed sensor can be seen in Figure 2. The entire sensor is 70 mm by 50 mm, without any miniaturization effort. The sensor was designed on the 1.6 mm thick FR4 with 17 µm of copper on both sides. The FR4 material has been chosen as substrate due to its availability and low cost. It has a relative permittivity of 4.4 and tan of 0.02 at 1 GHz frequency. On the top layer, a 50-Ω microstrip transmission line, 3 mm wide, was fabricated. A quarter wavelength stub with a length of 5 mm, and 3 mm wide was placed in the middle of the microstrip line. The copper of the FR4 was removed using a precise laser milling process (LPKF U3 system), while 1 mm off the tip of the stub was covered with glucose-sensitized MIP.

The same stub structure was built to use as a reference, without the MIP, to validate the measurements. The reference stub was constructed face to face with the sensing one to simplify the fabrication of the channel and flow of the aqueous solutions in the sensing region. It allows us to measure both responses simultaneously: the distance between two microstrips is 17 mm, and the tips of two stubs are 7 mm apart. Each microstrip was soldered to two subminiature version A (SMA) connectors as input and output RF ports. Two duplicated sensors from the same fabrication process were elaborated to validate the performance of the biosensor at several attempts.

The microfluidic system was designed, and 3D printed by a high-resolution SLA/DLP 3D printer (Creality Model LD-002R) to provide a specific volume of the solution in the sensing area. It was glued with transparent epoxy on the top of both stub resonators at the center to seal the sensing area and prevent leakage. To track the flow visually, a transparent photo resin was chosen to create the microfluidic system. It was printed with a length of 40 mm, a width of 15 mm, and a height of 4 mm. Two pairs of inlets and outlets were designed with a diameter of 2 mm and placed 18 mm apart. At the bottom of the housing, two channels were designed with a depth of 1 mm and a length of 18 mm to be filled with the glucose solutions to be characterized.

### 2.2. Simulation

**Figure 3:**
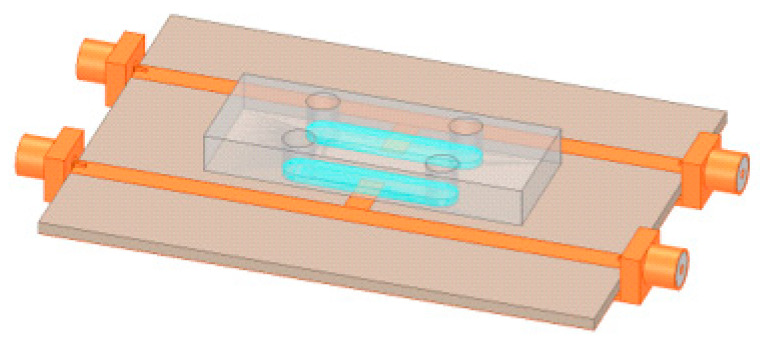
HFSS model of the uncoated microwave-based stub sensing device.

In order to analyze the performance of the sensor in terms of frequency responses, Ansys Electronics Desktop was utilized as a 3D EM simulator. The unloaded sensors were designed to operate at 7 GHz, and a shift towards lower frequencies was expected when the liquid under test flowed in the sensing region at the tip of the stub resonator due to the high relative permittivity of water $\varepsilon_{r}\;\sim \;80$. The modeled structure of an uncoated sensing device, shown in Figure 3, was used as a proof of concept for the RF design. Therefore, a set of simulations was conducted in which the flow channel was first unloaded, filled with air, and then loaded, filled with DI water with a relative permittivity of 80.

### 2.3. General Theory of Open Stub Resonator

**Figure 4:**
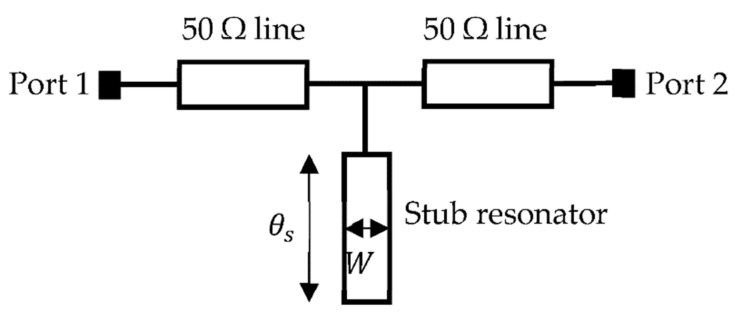
Open stub resonator illustration.

A general illustration of an open stub resonator is shown in Figure 4. The stub resonator is open-circuited at one end and connected in shunt with a 50-Ω transmission line at the other end.

The input impedance of the resonator was calculated as in Equation (1) which is extracted from the mathematical model of the open stub resonator [23].

(1) $$Z_{in}=-jZ_{s}\cot\theta_{s}=\frac{Z_{s}}{j\tan\theta_{s}}$$

where $Z_{s}$ is the characteristic impedance of the open stub resonator and $\theta_{s}$ is the electrical length in degre the ABCD matrix (Equation (2)) of the open stub resonator was extracted from Equation (1) shown below:

(2) $$\left[T_{s}\right]=\left[\begin{array}{cc} 1 & 0\\ Y_{in} & 1 \end{array}\right]=\left[\begin{array}{cc} 1 & 0\\ \frac{j\tan\theta_{s}}{Z_{s}} & 1 \end{array}\right]$$

Then, the S-parameters of the open stub resonator (*S*_12_, *S*_21_) were calculated by converting the ABCD matrix in Equation (2) to *S*-parameter in Equation (3). Hence,

(3) $$\begin{array}{ll} S_{12} & =S_{12}=\frac{2}{A+\frac{B}{Z_{0}}+CZ_{0}+D}\\ & =\frac{2}{1+\frac{0}{Z_{0}}+\left(\frac{j\tan\theta_{s}}{Z_{s}}\right)Z_{0}+1}\\ & =\frac{2}{2+\left(\frac{j\tan\theta_{s}}{Z_{s}}\right)Z_{0}} \end{array}$$

A normalized characteristic of impedance and impedance of resonator were considered $Z_{s}=Z_{0}=1\;$ to simplify analysis and discussion of the attenuation response of stub resonators. The electrical length of the stub should be regarded as $\theta_{s}$ = 90° (or π/2 radian) to achieve a notch response in an open stub resonator; hence, the *S*-parameter of Equation (3) simplifies in Equation (4),

(4) $$S_{12}=S_{21}=\frac{2}{2+\left(\frac{j\tan\left(\frac{\pi }{2}\right)}{Z_{s}}\right)1}\;\approx 0$$

or in decibel, as shown in Equation (5)

(5) $${\left|S_{12}\right|}^{2}dB={\left|S_{21}\right|}^{2}dB=20\;\log_{10}\left(0\right)=\infty \;dB$$

From Equation (5), an ideal infinite attenuation or notch is obtained when the electrical length of the open stub resonator is a quarter-wave (λ/4).

**Figure 5:**
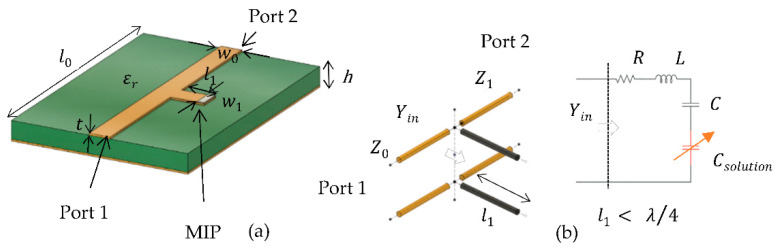
(**a**) Schematic illustration and (**b**) electrical equivalent model of the stub resonator with MIP.

Figure 5 illustrates the model of the biosensor with a stub as a quarter-wavelength resonator where the MIP was coated at the tip of the stub. An equivalent circuit model of our proposed sensor is shown in Figure 5b. The stub resonator can be modeled by a resistance (R), inductance (L), and capacitance (C) that is followed by capacitance representing solutions, shown as $C_{solution}$.

Therefore, the electrical length of the open stub resonator can be written as in Equation (6):

(6) $$\theta_{s}=2\pi fl_{res}\sqrt{LC_{total}}\;$$

and the length $l_{res}$ of the open stub resonator was calculated in Equation (7) as

(7) $$l_{res}=\frac{\lambda }{4}=\frac{1}{4f\sqrt{LC_{total}}}=\frac{c}{4f\sqrt{\varepsilon_{e}}}\;$$

where λ and f are the operating wavelength and frequency, respectively, $C_{total}$ is the total capacitance of C and $C_{solution}$, c is the speed of light in a vacuum, and $\varepsilon_{e}$ is effective permittivity is approximated in Equation (8) by

(8) $$\varepsilon_{e}=\frac{\varepsilon_{r}+1}{2}+\frac{\varepsilon_{r}-1}{2}\frac{1}{\sqrt{1+12\left(\frac{h}{W}\right)}}\;$$

which satisfies 1 <$\;\varepsilon_{e}$<$\;\varepsilon_{r}$ and depends on the relative permittivity ($\varepsilon_{r}$) and thickness (*h*) of the substrate, the conductor Width (*W*), and the frequency (*f*). The capacitance (C, $C_{solution}$) is a function of effective permittivity.

By changing the electromagnetic properties, such as the relative permittivity of the materials constituting or surrounding the resonator, the amount of energy stored in it and its losses can vary dramatically. To measure changes in permittivity due to varying concentrations of glucose, MIP was used as a selective receptor to trap target analytes, and microfluidic channels were incorporated in the sensor where the solutions flowed in, and a change in permittivity was sensed. The variation of concentrations of biomolecules attached to the MIP changes the permittivity, thus resulting in changes in the total capacitance and a shift in the resonant frequency according to Equation (7). The microfluidic device enables the water glucose solution to effortlessly pass the sensing area through the channels in the selected region where MIP was coated to study the changes in permittivity.

### 2.4. Reagents

In this research, all the chemicals utilized were analytical-reagent grade and acquired from Sigma Aldrich Co. (St. Louis, MI, USA) The chemicals used in this project are listed below:

- Acrylic acid (AA);
- Poly (methyl methacrylate) (PMMA);
- Ethylene glycol dimethacrylate (EGDMA);
- 4,4′-Azobis (4-cyanopentanoic acid) (ACPA);
- Dimethyl sulfoxide (DMSO).

### 2.5. Preparation of Proposed Sensor

**Figure 6:**
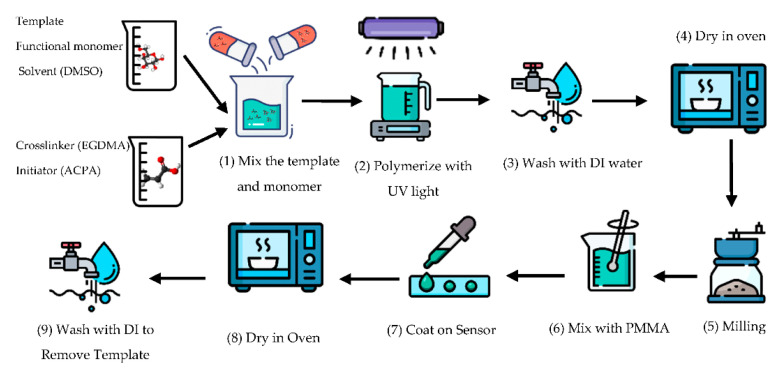
Illustration preparation of the sensor.

**Figure 7:**
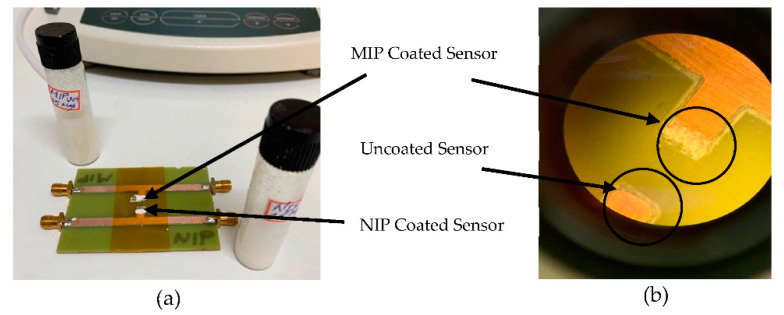
Photograph of (**a**) main fabricated biosensor with the stub resonators coated with MIP and NIP and (**b**) duplicate biosensor coated with MIP and uncoated under the microscope.

Both the MIP and the NIP were prepared by adapting procedures described in the literature [24,25]. The preparative steps are described below and illustrated in Figure 6:

(1) An amount of 1.0 mmol (69 µL) of AA, called a functional monomer, and 0.25 mmol (0.045 g) of glucose, used as a template, were dissolved in 5 mL DMSO, and stirred for 1 h. In another flask, 4.0 mmol (0.79 g) of EGDMA and 0.0050 g of ACPA were stirred for 1 h and then mixed with the former solution. Nitrogen gas was bubbled for 15 min to remove dissolved oxygen; (2) polymerization was initiated by exposure to UV light (wavelength of 385 nm and 405 nm) for 15 min; and (3) bulk polymers were washed with water to remove any non-polymerized monomers and solvents. (4) The MIP was dried by heating at 60 °C for 12 h and (5) then milled with mortar and pestle to make a fine powder. The same procedure was repeated to prepare the non-imprinted polymer (NIP); however, without adding the template (glucose) as a control to determine the selectivity of the MIP. (6) The polymer powders were mixed with a solution of 5% PMMA (Adhesion) in Acetone. The ratio of PMMA to MIP (or NIP) powders was 50:50. (7) A certain amount (20 µL) of the mixed solution was applied to the tip of the Stub resonator. Due to the rapid vaporization, a thin layer of MIP (or NIP) was formed on the sensor’s surface. (8) To evaporate the remaining solvent, the sensor was heated at 60 °C for 24 h. (9) The final step was to remove the template (glucose) from the MIP by washing the coated senor with DI water for 5 h. In order to deposit MIP and NIP accurately in the desired position (tip of the stub resonator) in sensors, a thin film of Kapton was utilized as a mask, shown in Figure 7a. The microscope image of the uncoated and the MIP-coated biosensor is illustrated in Figure 7b.

### 2.6. Glucose Sample Preparation and Measurement Procedure

A series of 5 water glucose solutions were prepared with glucose concentrations varying between 50 mg/dL and 400 mg/dL. The experiment was performed after the water glucose solutions rested for about 12 h to establish the anomeric equilibrium. The solutions were measured several times in both sensors (MIP-NIP and MIP-uncoated): right after resting, 1 h after the first measurement, the subsequent measurement was performed after 3 h of rest from the first attempt, and the last measurement was carried out after 24 h of rest. The glucose solutions were pumped one by one through the channel with the aid of a peristaltic pump. While the solution kept flowing continuously at a rate of 5 mL/min in each procedure, three sets of data were collected: right after injecting the solution on sensor, 5 min, and 10 min after the first measurement. After each measurement, DI water was pumped into the channel to wash it and remove absorbed glucose from the MIP. To avoid any shift in the dielectric properties of the glucose solutions, the temperature was maintained constant at 20 °C.

### 2.7. Measurement Setup

**Figure 8:**
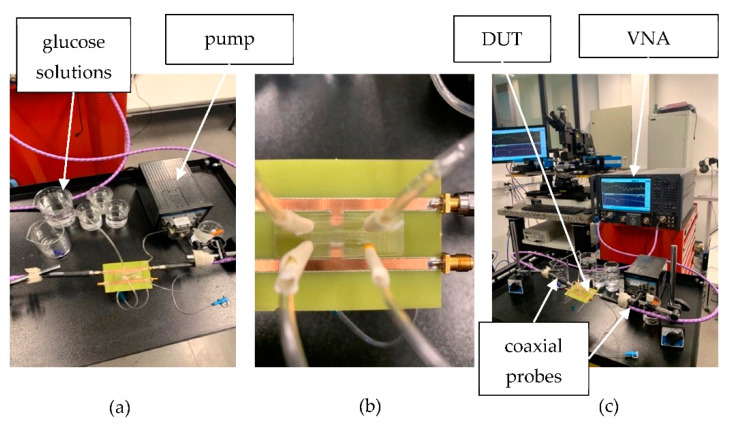
Photograph of (**a**) solutions and peristaltic pump, (**b**) assembled sensor, (**c**) experimental setup of the sensor with equipment.

The performance of the proposed biosensor was characterized in terms of its S-parameters using a performance vector network analyzer (VNA) (N5227B from, Keysight, Santa Rosa, CA, USA) in a two- ports configuration after the SOLT calibration up to 20 GHz. The measurement resolution is 0.1 dB, 1 Hz, and 0.5° in terms of magnitude, frequency, and phase, respectively. The device under test (DUT) was connected to the VNA ports via coaxial cables. All experiments were carried out under normal laboratory environment conditions. In this experiment, *S*_21_ scattering parameter was used to characterize the glucose concentration. Figure 8a and b show the fabricated sensing device with injection accesses, and Figure 8c shows the complete experimental setup for the measurement: VNA, DUT, coaxial probes, pump, and the solutions.

In the process, each time, first, the response of the sensor with an empty flow channel was measured, then the flow channel was filled with DI water continuously as a reference and also to wash the glucose from the MIP between each experiment to verify the reproducibility and repeatability of the study, and finally, filled with solutions with various glucose concentrations. The flow of the solutions was controlled using a pump.

## 3. Results

**Figure 9:**
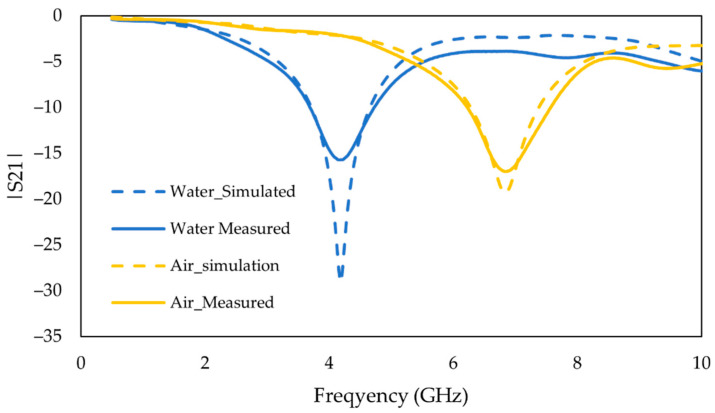
Measured vs. simulated frequency response of the insertion loss (*S*_21_) for flow channel filled with air and DI water.

The simulated and measured frequency response of the unloaded sensor (empty flow channel) (in yellow) and the measured frequency response of the loaded flow channel where DI passes the sensing area on top of the stub (in blue) compared to the simulation, up to 10 GHz, are demonstrated in Figure 9. The results in both experiments show the agreement between simulations and measurement is good for the uncoated sensor, which validates the simulation of the sensor’s behavior in Ansys.

**Figure 10:**
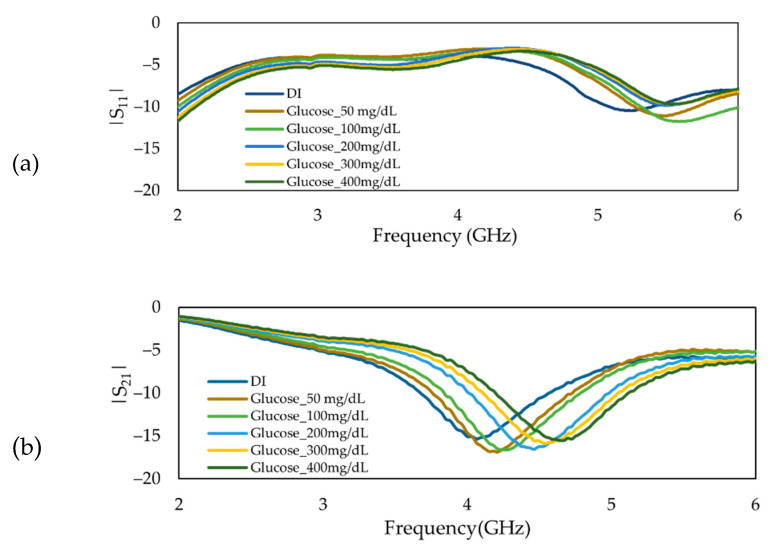
Frequency response of MIP-coated microwave-based stub biosensor (**a**) Reflection coefficient, *S*_11,_ and (**b**) Transmission coefficient, *S*_21_.

The reflection and transmission coefficients of the MIP-coated biosensor in the frequency range between 2 to 6 GHz are plotted in Figure 10. The sensor was exposed to glucose solutions with various concentrations ranging from 50 to 400 mg/dL. A shift towards higher frequencies can be observed in the absorption peak when the glucose content was increased in the solutions.

**Figure 11:**
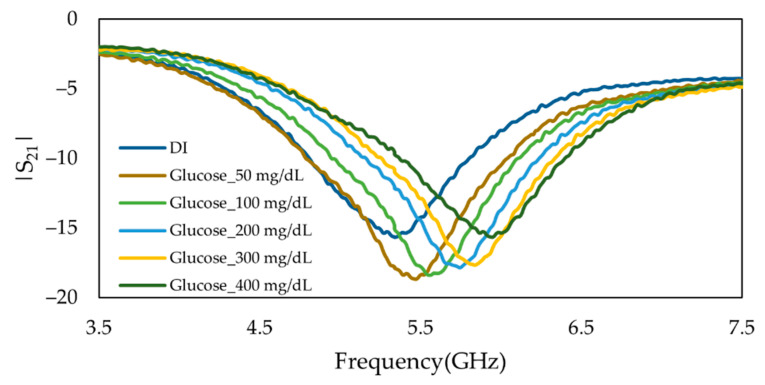
Measured frequency response of the insertion loss (*S*_21_) of duplicate MIP-coated microwave-based stub biosensor for the glucose solutions with concentration ranging from 50 mg/dL to 400 mg/dL.

In this study, we applied the fabricated MIP on two duplicated sensors to validate the performance of the biosensor at several attempts with different masses of MIP. These sensors were tested and the sensitivity in terms of a shift in the absorption peak due to changes in glucose concentrations was verified. Both main and duplicate sensors showed the same behavior in detecting different glucose concentrations in solutions which demonstrated the reproducibility of the sensor, as can be seen in the Frequency response of both main and duplicate senor in Figure 11.

**Figure 12:**
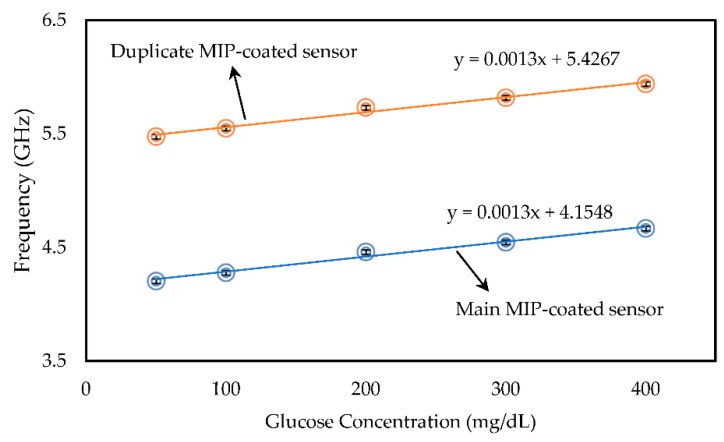
Calibration graph for the main and duplicate MIP-coated microwave-based biosensors exposed to glucose solutions.

The calibration curves of both main and duplicate MIP-coated sensors were constructed by plotting the absorption peak shift versus the glucose concentrations in Figure 12. In addition, the measurements were repeated several times for each sample solution and the results were presented using error bars. A linear relationship between the concentration of glucose and frequency shift can be seen in Figure 12, which confirms the behavior of the sensor has the same trend regardless of the mass of MIP.

**Figure 13:**
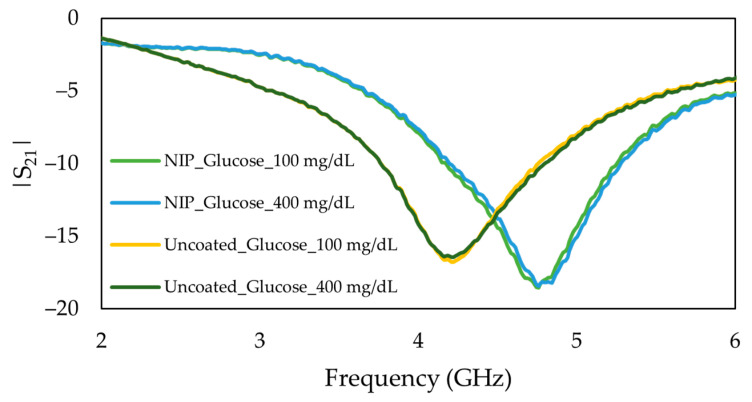
Frequency response of uncoated and NIP-coated microwave-based stub biosensor for the same glucose solutions of 100 mg/dL and 400 mg/dL.

In order to verify the proposed sensing principle, the procedure mentioned above was performed in uncoated and NIP-coated stub resonators. A set of measurements were performed using the same concentrations of glucose solutions, and the transmitted signal was recorded. As expected, the frequency response of the uncoated and NIP coated sensing devices, in the absence of the template cavities (glucose), did not change with the flow of the same glucose solutions of 100 mg/dL and 400 mg/dL in the flow channel, as demonstrated in Figure 13. This finding strongly indicates that measuring the various concentrations of glucose by utilizing a MIP coated biosensor increased the selectivity and, therefore, the detection of glucose levels.

**Figure 14:**
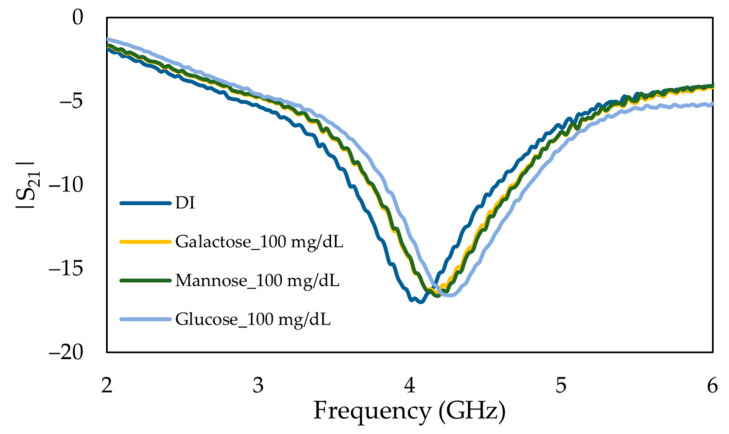
Measured frequency response of the insertion loss (*S*_21_) of main MIP-coated microwave-based stub biosensor exposed to 100 mg/dL of glucose, mannose, and galactose solutions.

To verify the selectivity of the MIP-coated sensor, solutions of glucose and two epimers of glucose, named mannose and galactose, were studied. Figure 14 shows the frequency response of 100 mg/dL of glucose, mannose, and galactose solutions. A slight shift in absorption peak can be observed due to the changes in the relative permittivity of the sample solutions.

**Figure 15:**
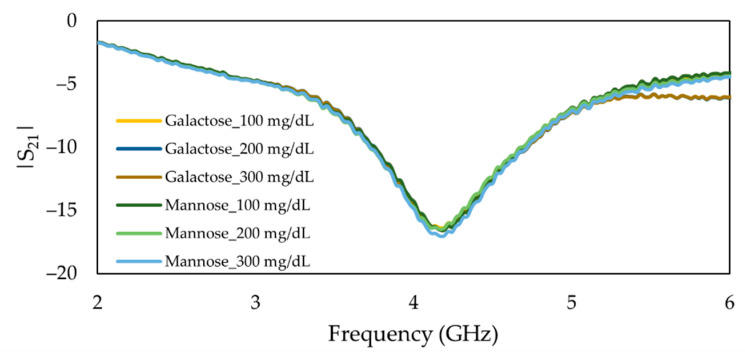
Measured frequency response of the insertion loss (*S*_21_) of main MIP-coated microwave-based stub biosensor exposed to mannose and galactose solutions ranging from 100 mg/dL to 300 mg/dL.

The experiment was followed by the flow of the mannose and galactose solutions ranging from 100 mg/dL to 300 mg/dL in the channel. The frequency response of the MIP-coated sensor did not change with the flow of the solutions, even by increasing the concentration of the mannose and galactose, as demonstrated in Figure 15. This evidenced that our proposed sensor is effectively selective toward glucose than its epimers.

**Figure 16:**
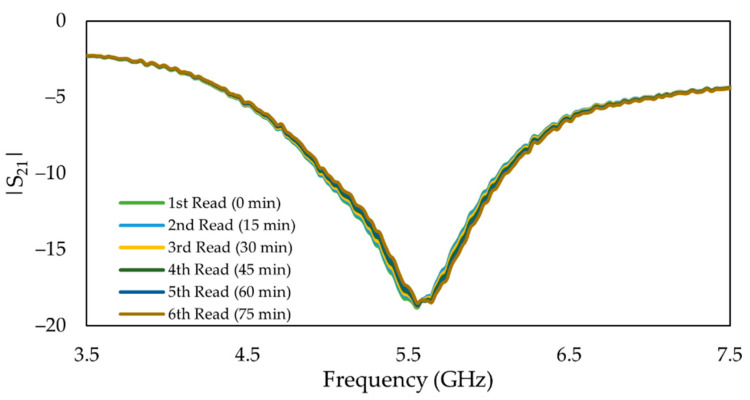
Repeatability and stability test for frequency changes in the duplicate MIP-coated microwave-based stub biosensor: Frequency response exposed to 100 mg/dL glucose solution.

Furthermore, to examine the repeatability and stability of the responses, the 100 mg/dL glucose solution was pumped continuously into the flow channel for 75 min, and the transmitted signal was recorded at the interval of 15 min. The results on Figure 16 show the repeatability and stability of the duplicate MIP-coated sensor with respect to the changes in glucose concentrations in terms of the shift in the resonant frequency (absorption peak). A relative standard deviation (R.S.D.%) of 4.61 was obtained. This indicated that the sensor performs in a repeatable manner which is comparable with the previously reported values of 3.9–5.6% [20]. As expected, the frequency response does not change during the time.

Although the studied range of glucose concentrations (50–400 mg/dL) covers most of the glucose levels in the human body, the linear response of the proposed biosensor confirmed that solutions with various concentrations can easily be measured after appropriate dilution. Table 1 shows the summary of analytical parameters of the proposed biosensor in the studied range of glucose concentrations. To understand the extent of imprinting and explicitness of prepared MIP, the imprinting factor was presented in Equation (9), which was calculated by dividing the frequency shift response of MIP ($∆f_{MIP}≅\;134\;\mathrm{MHz}$ from Figure 10b) by the frequency shift response of NIP ($∆f_{NIP}≅\;12\;\mathrm{MHz}$ from Figure 13). In proposed MIP-coated glucose sensor, the imprinting factor of MIP is 11 times higher than that of NIP.

(9) $$IF=\frac{∆f_{MIP}}{∆f_{NIP}}=11\;$$

## 4. Discussion

Blood glucose level measurement is a challenging task, and there are many factors affecting measurement accuracy that need to be considered when a real-life glucose range measurement is concerned. Table 2 summarizes the experimental results of glucose measuring sensors using planar transmission lines stated in the literature and that of our proposed sensor. The studied frequency and concentration ranges and the average sensitivity values with respect to the concentration range for each sensor are also presented in this table. Almost any sensor in the literature shows a frequency shift for different concentrations of glucose; however, most of them investigate unrealistic glucose levels. Indeed, in most cases, including [11], researchers eliminate the biomarker to measure glucose level. However, the result may not be suitable for real-life blood glucose level measurement since they ignore the effect of other physiological parameters and bioanalyses; hence their selectivity is ambiguous. On the other hand, the enzyme base biomarkers, as in [13,14], give certain confidence that the results are related to the glucose concentration. Still, they are not real-time and less sensitive due to the chemical reaction between glucose and enzyme and insufficient stability. Research shows the relative shift of frequency in the presence of GOD occurs within 25 min for glucose to reach the saturation level. 

The proposed MIP-coated open stub resonator biosensor is developed as an inexpensive and sensitive sensing device that detects variation in glucose concentration in the solution by measuring shifts in the absorption peak. Acrylic acid (AA) is selected as a suitable functional monomer, and 4,4′-Azobis (4-cyanopentanoic acid) (ACPA) is chosen as a fast initiator. Furthermore, the frequency response of the stub resonator shifts due to the varying glucose concentrations, which demonstrates an excellent linear correlation (R^2^ = 0.9839) within the range of 50–400 mg/dL, while the lower limit of detection (LLD) of the sensor is discovered to be 2.4 ng/dL. Our sensing device differentiates the glucose variation regardless of the mass of MIP and is effectively selective toward glucose compared to similar compounds. In addition, it requires a small volume of samples (40 µL) and is highly sensitive (1.3 MHz/(mg/dL)) compared to other similar approaches. This capacity will help us to miniaturize the sensor. Moreover, the combination of molecularly imprinted technology and a microwave resonator that can be fabricated easily at a low cost, provides the opportunity to make a passive and battery-less sensing device for point-of-care monitoring purposes.

## Figures and Tables

**Table 1:** Analytical parameters of sensing device coated with MIP.

^a^ R^2^	Sensitivity Factor (MHz/mg dL^−1^)	^b^ LLD (ng dL^−1^)	^c^ RSD%	Imprinting Factor
0.9839	1.3	2.4	4.61	11

^a^ Square Correlation Coefficient, ^b^ Lower Limit of Detection, ^c^ Relative Standard Deviation.

**Table 2:** Comparison with the state-of-the-art the MIP-coated microwave-based biosensor.

Ref.	Sensor Type	Sample Volume (µL)	Glucose Concentration (mg/dL)	Nonloaded $f_{r}$ (GHz)	Operating $f_{r}$ (GHz)	Sensitivity $f_{r}$ (MHz per mg/dL)	R^2^	Remarks
	Single SRR	90 μL	0–50,000	1.83	1.49	0.107 × 10^−2^	0.79	Enzyme-coated
	Single SRR	90 μL	0–40,000	1.82	1.59	0.174 × 10^−2^	0.93	Enzyme-coated
	MLIN	16 0μL	100–300 1000–8000	-	4.8–5.7	32 × 10^−2^ 3.2 × 10^−2^	0.98	Non-Enzyme
**This work**	**Stub Resonator**	**40 μL**	**0–400**	**7**	**4.4**	**1.3**	**0.9839**	**MIP-coated**
